# A *ZFYVE19* gene mutation associated with neonatal cholestasis and cilia dysfunction: case report with a novel pathogenic variant

**DOI:** 10.1186/s13023-021-01775-8

**Published:** 2021-04-14

**Authors:** Claudia Mandato, Maria Anna Siano, Lucia Nazzaro, Monica Gelzo, Paola Francalanci, Francesca Rizzo, Ylenia D’Agostino, Manuela Morleo, Simona Brillante, Alessandro Weisz, Brunella Franco, Pietro Vajro

**Affiliations:** 1 Department of Pediatrics, Santobono-Pausilipon Children’s Hospital, Naples, Italy; 2 Postgraduate School of Pediatrics Department of Medicine, Surgery and Dentistry “Scuola Medica Salernitana”, University of Salerno, Via Allende, 84081 Baronissi, (SA) Italy; 3 Pediatric Clinic, “SS. Giovanni Di Dio and Ruggi D’Aragona” University of Salerno Hospital, Via San Leonardo, 84131 Salerno, Italy; 4 Department of Molecular Medicine and Medical Biotechnology, Faculty of Medicine, University of Naples Federico II, Naples, Italy; 5 Pathology Unit. Department of Laboratories, IRCCS Bambino Gesù Pediatric Hospital, Rome, Italy; 6 Medical Genomics Program, “SS. Giovanni Di Dio and Ruggi D’Aragona” University of Salerno Hospital, Salerno, Italy; 7 Laboratory of Molecular Medicine and Genomics, Department of Medicine, Surgery and Dentistry “Scuola Medica Salernitana”, University of Salerno and Genome Research Center for Health (CRGS), Baronissi, (SA) Italy; 8 Telethon Institute of Genetics and Medicine (TIGEM), Pozzuoli, Naples Italy; 9 Medical Genetics, Department of Medical Translational Science, Faculty of Medicine, University of Naples “Federico II”, Naples, Italy

**Keywords:** Cholestasis, Ciliopathy, ZFYVE19, Children

## Abstract

**Background:**

ZFYVE19 (Zinc Finger FYVE-Type Containing 19) mutations have most recently been associated to a novel type of high gamma-glutamyl transpeptidase (GGT), non-syndromic, neonatal-onset intrahepatic chronic cholestasis possibly associated to cilia dysfunction. Herein, we report a new case with further studies of whole exome sequencing (WES) and immunofluorescence in primary cilia of her cultured fibroblasts which confirm the observation.

**Results:**

A now 5-year-old girl born to clinically healthy consanguineous Moroccan parents was assessed at 59 days of life due to severe cholestatic jaundice with increased serum bile acids and GGT, and preserved hepatocellular synthetic function. Despite fibrosis/cirrhosis and biliary ducts proliferation on liver biopsy suggested an extrahepatic biliary obstacle, normal intra-operatory cholangiography excluded biliary atresia. Under choleretic treatment, she maintained a clinically stable anicteric cholestasis but developped hyperlipidemia. After exclusion of the main causes of cholestasis by multiple tests, abnormal concentrations of sterols and WES led to a diagnosis of hereditary sitosterolemia (OMIM #618666), likely unrelated to her cholestasis. Further sequencing investigation revealed a homozygous non-sense mutation (p.Arg223Ter) in ZFYVE19 leading to a 222 aa truncated protein and present in both heterozygous parents. Immunofluorescence analysis of primary cilia on cultured skin fibroblasts showed a ciliary phenotype mainly defined by fragmented cilia and centrioles abnormalities.

**Conclusions:**

Our findings are consistent with and expands the recent evidence linking ZFYVE19 to a novel, likely non-syndromic, high GGT-PFIC phenotype with neonatal onset. Due to the possible role of ZFYVE19 in cilia function and the unprecedented coexistence of a coincidental hereditary sterol disorder in our case, continuous monitoring will be necessary to substantiate type of liver disease progression and/or possible emergence of a multisystemic involvement. What mentioned above confirms that the application of WES in children with undiagnosed cholestasis may lead to the identification of new causative genes, widening the knowledge on the pathophysiology.

**Supplementary Information:**

The online version contains supplementary material available at 10.1186/s13023-021-01775-8.

## Background

Cholestasis affects approximately 1 in every 2500 term infants and it is defined as reduced bile formation or flow resulting in the retention within the liver of biliary substances normally excreted into bile and destined for elimination into the intestinal lumen. Neonatal cholestasis may stem from several conditions requiring either medical or surgical treatment. Some forms may remain of undetermined origin (so called, with descriptive term, “idiopathic neonatal hepatitis”) [1]. In a European study, biliary atresia was the most common diagnosis (41%), followed by progressive familial intrahepatic cholestasis (PFIC) and idiopathic cases (approximately 10% each), which thus represent a minor but still challenging and substantial group of disorders [2]. Advanced sequencing methods promise to further increase the diagnostic yield of genetic approaches. [3, 4].

While obstacles on the extrahepatic biliary tree [e.g. surgical emergencies such as Biliary Atresia (OMIM #210500)] are quite regularly characterized by high levels of gamma-glutamyl transpeptidase (GGT), assessment of intrahepatic cholestasis is more difficult. In this respect, patients can be usefully categorized by whether levels of serum GGT remain in normal ranges despite hyperbilirubinemia activity or rise together with serum concentrations of conjugated bilirubin [1]. High GGT values often reflect rare genetic cholangiopathies such as Alagille syndrome (OMIM #118450), Renal Cysts and Diabetes Syndrome (RCAD) (OMIM #137920), alpha-1-antitrypsin deficiency (OMIM #107400), *ABCB4* disease (OMIM #171060, Phenotype MIM number 60234), and neonatal sclerosing cholangitis (OMIM #61739) (involving the *CLDN1, DCDC2, KIF12*, or *PPM1F* genes respectively). Recently, Luan et al. described a new and previously unidentified genetic cause, the zinc finger FYVE-type containing 19 (*ZFYVE19*), alias abscission/nocut checkpoint regulator, as a novel cause of high-GGT infantile cholestasis in a small series of Chinese children with a DCDC2-unrelated neonatal sclerosing cholangitis-like phenotype (Congenital Hepatic Fibrosis and Sclerosing Cholangiopathy) [5]. The *ZFYVE19* protein controls cytokinesis and resides mainly on centrosomes in interphase and early mitosis (https://www.genecards.org/cgi-bin/carddisp.pl?gene=ZFYVE19). In post-mitotic cells, centrosomes move to the apical cell surface and contribute to the formation of primary cilia. Epithelial cells lining the lumen of intrahepatic bile ducts, i.e. cholangiocytes, display primary cilia consisting of (a) the microtubule-based axoneme that has nine peripheral microtubule doublets arranged around a central core that does not contain two central microtubules, and (b) the basal body, from which the axoneme emerges. Primary cilia are well established sensory organs and their physiologic significance in cholangiocytes remains unclear. Cholangiocyte cilia extending from the apical plasma membrane to the lumen of the bile duct are ideally positioned to detect changes in bile flow, composition and osmolality. These mechanosensory, osmosensory and chemosensory organelles have been proposed to control cholangiocytes’ functions such as the formation of ductal bile [6]. Interestingly, biliary specimens in chronic cholestasis due to syndromic and non-syndromic biliary atresia may show shorter cilia, abnormal in their orientation, and less abundant compared to controls. While this may result from the same severe cholestasis or inflammation themselves, it has been suggested that it may also reflect common mechanistic pathways in different forms of biliary atresia and may have implications for understanding the progression of the disease [7].

Herein we report another patient with cholestasis due to a novel nonsense homozygous pathogenic variant of the *ZFYVE19* gene inherited from the heterozygous parents, and further investigate the possible underlying cholestatic pathomechanism.

## Materials and methods

### Participant

DB is now a 5-year-old girl, only child, with neonatal-onset PFIC, born at term at 38 weeks to consanguineous, clinically healthy, Moroccan parents. History of maternal hypertransaminasemia at the 7th month of pregnancy. Assessed at 59 days of life due to jaundice, laboratory data showed cholestatic jaundice with increased bilirubin (total 11.5 mg/dl; conjugated 6.8 mg/dl), total bile acids (150 µmol/L), transaminases [Glutamic Oxaloacetic Transaminase (GOT, 365 U/L), Glutamic Piruvic Transaminase (GPT, 248 U/L) and GGT (1791 U/L)], mild hyperlipidemia (Total Cholesterol 158 mg/dl). Hepatocellular synthetic function (Albumin 3.2 g/dl; Prothrombin Time 100%; pCHE 3999) was preserved (see Additional file 1).

**Fig. 1:**
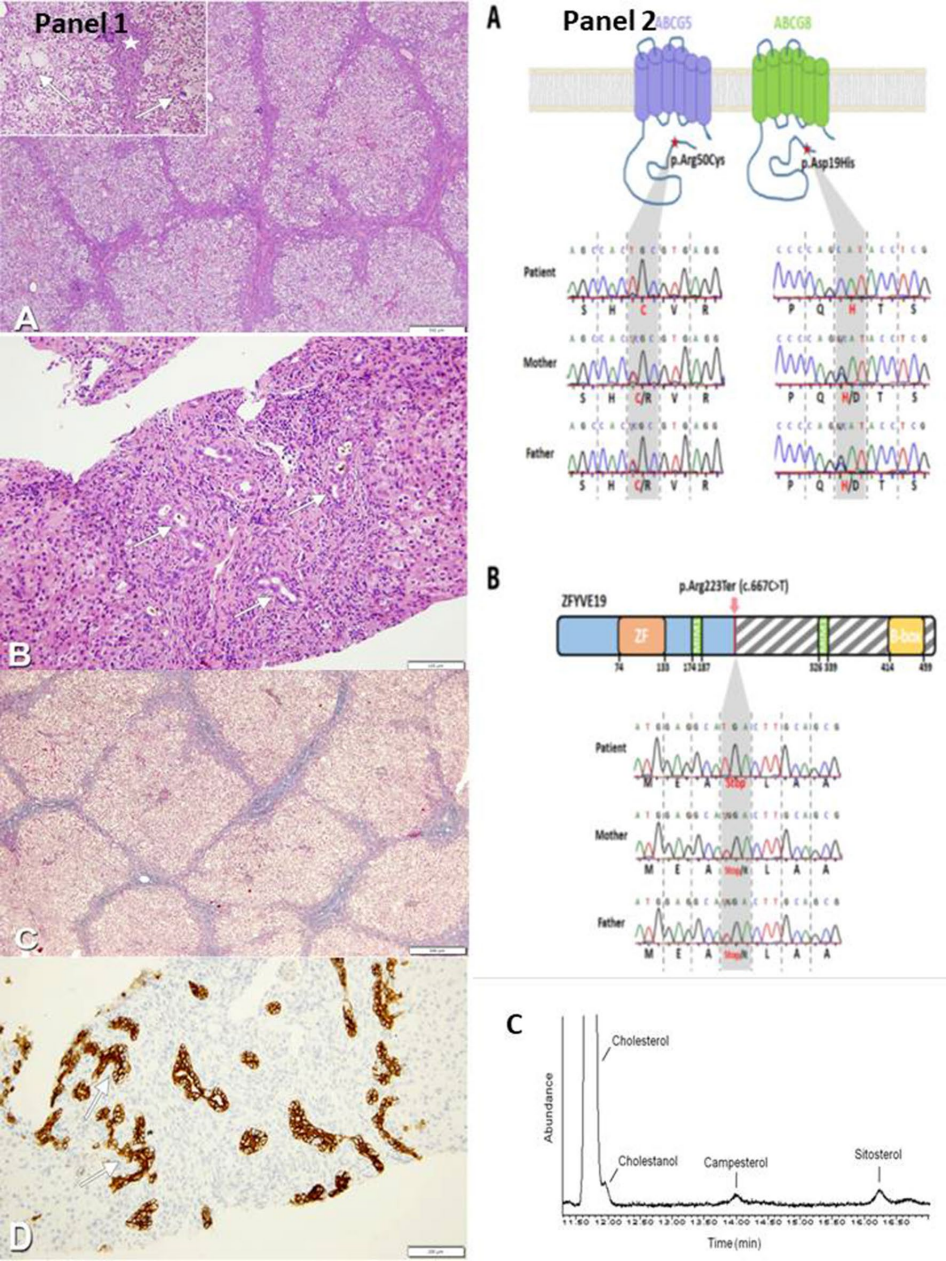
Panel 1 Liver Histology. **a** Liver histology shows a cirrhotic aspect (HE, 4×). Giant multinucleated hepatocytes (arrows) and extramedullary erythropoietic foci are observed (star) (inset, HE, 10×). **b** In fibroedematous portal tracts dense inflammatory infiltration and biliary ducts proliferation (arrows) could be detected (HE, 20×). **c** Fibrous and edematous septa of micronodular cirrhosis (Masson trichromica, 4×). **d** Immunohistochemistry with anti-CK7 identifies dysmorphic neoduttules mimicking ductal plate malformation (arrows) (CK7, 20×). Panel 2 Mutations identified in ABCG5, ABCG8 and ZFYVE19 genes by WES and Sanger. **a** Upper panel: schematic structure of ABCG5 and ABCG8 transporters with mutations [ABCG5 p.Arg50Cys exon 2/13, c.148C > T (rs6756629) and ABCG8 p.Asp19His exon 1/13, c.55G > C (rs11887534)] indicated by a red star. Lower panel: results of Sanger sequencing of ABCG5 and ABCG8 mutations, non-synonymous mutations were homozygous in the patient and heterozygous in the two parents (grey boxes highlighting the amino acids influenced by these nucleotide mutations). **b** Upper panel: domain structure of ZFYVE19 protein showing the non-sense mutation [ZFYVE19, p.Arg223Ter exon 5/11, c.667C > T (rs375497733)] identified that introduces a premature stop codon leading to a 222 aa truncated protein compared to the 471 aa wild type one. Lower panel: results of Sanger sequencing of ZFYVE19 mutation; non-sense mutations were homozygous in the patient and heterozygous in the two parents (grey boxes highlighting the amino acids influenced by these nucleotide mutations). **c** Gas chromatography–mass spectrometry and flame ionization detector profile of sterols extract from patient's plasma

As clinical (hypocholic stools, enlarged liver with increased consistency), laboratory (high GGT values) and liver biopsy (fibrosis/cirrhosis and biliary ducts proliferation suggestive of an obstacle on the extrahepatic biliary tree) (Fig. 1, panel 1a–d) data were compatible with biliary atresia, surgical intervention was planned. Intraoperatory cholangiography, however, was normal and clearly excluded it. As per our institution protocol, [1, 8] main causes of infectious, endocrine, and structural cholestasis were ruled out by appropriate tests. Since common genetic-metabolic conditions were ruled out also by testing of an extended liver panel (including analysis of *ABCB4, DCDC2* and other high GGT cholestasis associated genes), whole exome sequencing (WES) was therefore requested.

Patient’s family consent was obtained prior to genetic testing. All information in this report has been de-identifed in accordance with HIPAA and institutional review board regulations.

### Genetic study

Mutations were identified through WES and targeted Sanger sequencing.

- Whole exome sequencing

Blood samples were collected from the patient and her parents and genomic DNA (gDNA) was isolated from peripheral blood leukocytes. WES was performed using a trio-based approach (patient, mother and father) as previously described [9]. In brief, 200 ng of gDNA was used for library preparation and exons were captured using the SureSelect Human All Exon v6 kit (60 Mb; Agilent Technologies) and sequenced on Illumina NextSeq500 with 75 bp paired-end reads. Sequence reads were demultiplexed obtaining ~ 78, 79, 61 million reads for the patient, father and mother, respectively, and quality of the sequencing was evaluated through the FastQC program. The raw reads were aligned to human genome assembly GRCh37 (hg19) using BWA enrichment (v2.1.2). Variants were identified using the Illumina DRAGEN (Dynamic Read Analysis for GENomics) Bio-IT Platform (v3.2.8) and annotated using GeneTalk (GmbH Berlin, Germany). Variants were filtered under an autosomal-recessive model of inheritance and stop, splice, frameshift and non-synonymous variants with prevalence < 1% in the 1000 Genomes Project and Exome Sequencing Project were retained.

- Sanger sequencing

Candidate variations were confirmed by Sanger sequencing using the following primers: for ABCG8, 5′-tccccagagtggcttcagttg-3′ (fwd) and 5′-acactgcttgatgtccgggt-3′ (rev); for *ABCG5*, 5′-gtgaaagaatgcagggacagc-3′ (fwd) and 5′-atcaaacctgtggctttcttgtt-3′ (rev); for *ZFYVE19*, 5′-gaaacagtgagtgggtgcct-3′ (fwd) and 5′-cctgtatctgggcttctgctg-3′ (rev). A standard PCR protocol was used with FastStartTaq DNA Polymerase kit (Roche Life Science, Indiana, USA) and sequencing was carried out at Eurofins Genomics (Ebersberg, Germany).

### Cilia analysis

Cells were grown on glass coverslips pretreated with poly-lysine (Sigma-Aldrich) to facilitate attachment of cells in 24-well plates and cultured in Dulbecco’s Modified Eagle Medium (DMEM, Gibco) supplemented with 20% FBS, 1 mM l-glutamine, and 1% antibiotics (penicillin/streptomycin). When cells attained 90% confluence, they were cultured in serum-free media for another 48 h to induce ciliogenesis as described [10, 11]. Cells were fixed with ice-cold methanol for 5 min, then permeabilized and immunostained with antibodies against the ciliary component ADP-ribosylation factor-like GTPase 13B (ARL13B; rabbit polyclonal antibody, 17711-1-AP, 1:1000 dilution; Proteintech) and the centriole marker γ-tubulin (T6557, 1:2000 dilution; Sigma-Aldrich). Donkey anti-rabbit AlexaFluor 488 (A21206) and donkey anti-mouse Alexa Fluor 568 (A21202) from Thermo Scientific were used as secondary antibodies both at 1:1000 dilution. Nuclei were stained with Hoechst (33342, Sigma).

Samples were examined under LSM800 High-resolution confocal laser‐scanning microscopes (Zeiss). Optical sections were obtained under a X40 oil-immersion objective at a definition of 1024 X 1024 pixels, adjusting the pinhole diameter to 1 Airy unit for each emission channel. Percentages of ciliation and of centriolar abnormality in each cell group (n > 300) were determined in 2 independent experiments, and significance was determined with paired t-testing.

## Results

### WES

Investigation by WES performed after informed consent for genetic testing revealed homozygous mutations in the *ABCG5* and *ABCG8* genes [*ABCG5* p.Arg50Cys exon 2/13, c.148C > T (rs6756629) and *ABCG8* p.Asp19His exon 1/13, c.55G > C (rs11887534)] (Fig. 1, panel 2a). In this patient mutations were also found in the *ZFYVE19* transcript which is ubiquitously expressed. Nonsense mutations [p.Arg223Ter exon 5/11, c.667C > T (rs375497733)] were homozygous in the patient, and heterozygous in the two parents, leading to a 222 aa truncated protein compared to the 471 aa wild type protein (Fig. 1, panel 2b).

### Cilia analysis

**Fig. 2:**
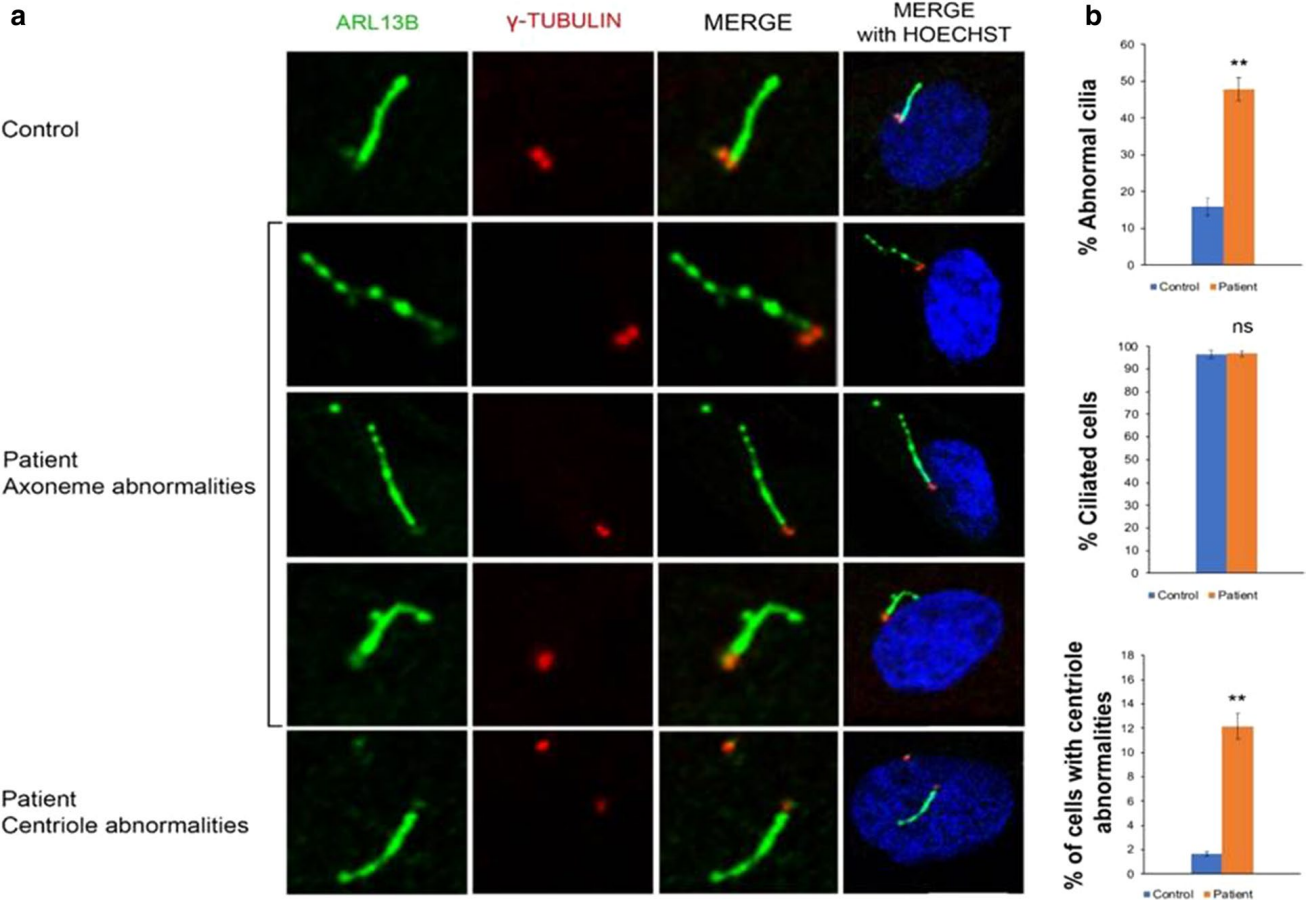
Cilia-related phenotypes. **a** Top panel control fibroblasts. Middle and bottom panels, representative images of fibroblasts derived from patient. Cells were starved for 48 h. Centrioles and cilia were labeled with anti γ-tubulin (red) and ARL13B (green) antibodies, respectively. Hoechst labels nuclei (blue). Scale bar: 10 μm. **b** Histograms show the percentage of fibroblast cells with abnormal cilia/basal bodies/centrioles (top), the percentage of ciliated cells (middle), and the percentage of cells with centriolar abnormalities (bottom), in comparison with the normal control. ≥ 200 cells analyzed per sample. Data are expressed as mean values; error bars indicate the SEM. Paired Student’s t-test were applied. **p ≤ 0.01 ns = not significative

Given the recent findings [5], we next investigated ciliogenesis on patient’s cultured skin fibroblasts. Immunofluorescence analysis was performed using an antibody against ARL13B that stains the axoneme of primary cilia and γ-tubulin that decorates centrosomes and centrioles. The total number of ciliated cells appeared comparable to that of control fibroblasts. However, analysis of cilia morphology revealed a significantly increased number of cilia with non-canonical ciliary structure in fibroblasts obtained from patient (about 50% of cilia) compared to controls (about 17%): the ciliary phenotype was mainly defined by a discontinuous axoneme, marked by gaps in ARL13b staining in all z-planes. Moreover, a significant number of cells showed centriolar abnormalities resulting from extra centrioles or increased distance between the two centrioles. (Fig. 2a, b).

### Plasma sterols analysis

Plasma sterols analysis by gas chromatography (GC)-mass spectrometry (MS) and flame ionization detector (FID) showed an increase in cholestanol (1.44 mg/dl–unv < 0.71), campesterol (0.89 mg/dl–unv < 0.33) and sitosterol (1.44 mg/dl–unv < 0.39) levels in agreement with the diagnosis of Sitosterolemia (OMIM #618666), a condition which appeared, however, unrelated to her cholestatic liver disease (Fig. 1, panel 2c).

### Follow-up

Bilirubin normalized with time but GGT continued to be elevated.

Under ursodeoxycholic acid (UDCA) and rifampin treatment (doses ranging between 20–28 mg/kg/day and 2–5 mg/kg/day, respectively), the patient has now a clinically stable cholestatic liver disease characterized by splenomegaly, persisting hepatomegaly, normalized bilirubin and satisfactory hepatocellular synthetic function, but increasing hyperlipidemia, high serum bile acids and hepatobiliary enzymes (Additional file 1: Table 1). Intra and extrahepatic bile ducts at two recent cholangio-magnetic resonance imaging analyses continue to appear within normal limits. Other organs and systems including renal function and neurological exam and psychomotor development, are within normal limits.

A recent plasma sterols follow-up study after starting a low sterols diet revealed a slight reduction of cholestanol levels (− 21.5%), although the measured concentration was higher compared to the reference value. On the other hand, campesterol and sitosterol levels were slightly increased (22% and 9%, respectively), compared to the first analysis (July 2019).

## Discussion

Our study offers a first confirmation of the link between ZFYVE19 and a neonatal-onset chronic cholestasis phenotype as well as further experimental evidence for a cilia involvement in this condition. It also endorses the concept that the rate of patients diagnosed with the descriptive term ‘‘idiopathic neonatal hepatitis’’ will continue to decline with the advancement in diagnostic evaluation and the use of next-generation DNA sequencing technologies [1, 3, 4]

Our patient with a high GGT cholestatic hepatobiliary disease was erroneously suspected of having extrahepatic biliary atresia on the basis of the clinical, laboratory and liver histological pictures. Subsequently, only WES analysis clarified the nature of the disorder by detecting mutations in the protein coding gene *ZFYVE19*, a putative key regulator of abscission step in cytokinesis, i.e., the very last phase of mitotic cell division that results in a physical separation of two daughter cells (https://www.genecards.org/cgi-bin/carddisp.pl?gene=ZFYVE19). This transcription unit has hitherto been described as a novel cause of high-GGT infantile cholestasis only in a series of 9 Chinese children with neonatal sclerosing cholangitis-like phenotype. On histopathologic study those patients had portal-tracts widening and fibrosis without interface activity, and ductular reaction consonant with a persistence of the ductal plate [5]. Both these histological features are in keeping with those of the portal tract of the liver biopsy of our patient (Fig. 1, panel 1a–d) and also of other patients with mutations in other genes encoding cilium-associated proteins. More specifically, the renal-hepatic ciliopathy due to Doublecortin domain-containing protein 2 (DCDC2) mutations results in a hepatic histological pattern resembling congenital hepatic fibrosis, but high GGT cholestasis in this condition appears independent from biliary ducts infections but rather is linked to sclerosing cholangitis. Other high GGT putative cholangio-ciliopathies caused by mutations in Kinesin family member 12 (*KIF12*), and protein phosphatase 1F (*PPM1F*) also have histopathologic features suggestive of biliary cirrhosis ± paucity of bile ducts and liver fibrosis [12]. Finally, variants in *ANKS6* (Ankyrin Repeat and Sterile Alpha Motif Domain Containing 6), which encodes a protein that interacts with other proteins of the Inv compartment of cilium (*NEK8, NPHP2/INVS,* and *NPHP3*), were detected in other patients who had histological periportal fibrosis and cholestatic hepatopathy along with a picture of infantile nephronopthisis-related ciliopathy (NPHP-RC) [13, 14].

In the Chinese study of Luan*, ZFYVE19* depletion in retinal pigmented epithelial cell line (hRPE1) showed supernumerary centrioles and separation/abnormal arrangement of the centriole pair(s). However, ciliogenesis was not affected and additional cilia protrude from supernumerary basal bodies/centrioles. Similar ciliary and centrioles phenotypes were observed in *ZFYVE19*-deficient fibroblast-like cells derived from patient iPSCs [5]. Our study on skin fibroblasts from the *ZFYVE19*-mutated patient confirmed both the centrioles abnormalities and the comparable number of ciliated cells observed between the patient’s sample and control, as described in Luan et al. Moreover, we observed that the ciliary phenotype was also characterized by discontinuous staining of ARL13b in primary cilia axonemes. The differences observed in cilia morphology may be explained by the different mutations studied or by the different experimental conditions as our study was conducted on primary fibroblasts while the Chinese study was performed in *ZFYVE19*-knockdown hRPE1 cells and patient-derived iPSCs [5]. Our results, however, confirm the observation of cilia dysfunction in *ZFYVE19-*mutated patients also in cells not subjected to chronic cholestasis. The abnormalities observed in patient-derived skin fibroblasts reflect the cilia abnormalities of the affected tissues and indicate that indeed *ZFYVE19* plays a role in ciliogenesis. Conversely, the eventual absence of a ciliary phenotype in fibroblasts would have not ruled out a tissue-specific ciliary defect [15]. Table 1 summarizes the main findings in the Chinese series and our case. Out of a cohort of 25 children with undiagnosed high-GGT cholestasis, 9 showed *ZFYVE19* mutation. Only in one parental consanguinity had been established, as in our case. Similarly, no child was icteric at enrolment in the study although four patients presented with neonatal jaundice. GGT, GOT, and GPT were elevated in all patients. Seven were treated with UDCA and 4 required liver transplantation.

**Table 1:** Main findings in the 9 Chinese patients and our case

Reference	Total cases	Sex, Age onset	ZFYVE19 gene mutations		First clinical features	Clinical evolution	Hystopathologic features	Renal cystic change	↑GGT ↑GPT ↑GOT	Outcome	Ciliary studies
			Nucleotide change	Aminoacid change							
Luan et al. (2020)	9	M, birth	c.314C > G	p.S105X	Neonatal jaundice	Resolved Jaundice, 8 months. Hepato-splenomegaly. UGIH	Explanted liver, micronodular cirrhosis, ductular reaction	No	Yes	LT at 5 y 6 m	In ZFYVE19-knockdown hRPE1 cells, a prominent phenotypical abnormality, was an increase in numbers of basal bodies/centrioles. Separation/abnormal arrangement of the centriole pair(s) was also observed. However, cilium assembly was not affected and extra cilia took shape at extra basal bodies/ centrioles In ZFYVE19-deficient fibroblast-like cells derived from patient iPSCs, similar phenotypes involving abnormalities of ciliary and centriolar numbers but not of cilium assembly were demonstrated In both cells: supernumerary centrioles and cilia when ZFYVE19 was depleted
		*Sisters* F, 5 y	c.226A > G c.314C > G	p.M76V p.S105X		Hepato-splenomegaly. Portal hypertension	DPM	No	Yes	Improved LFTs on UDCA at 15 y worsened on UDCA at 17 y	
		F, 14 m			Hepatomegaly	Hepatomegaly		No	Yes	Improved LFTs on UDCA at 10 y 4 m; UDCA stopped at 12 y 4 m	
		M, 40 days	c.314C > G c.514C > T	p.S105X p.R172X	Neonatal jaundice, diarrhoea	Pruritus. Hepato-splenomegaly. Portal hypertension	DPM	No	Yes	Improved LFTs on UDCA at 14 y 1 m	
		M, 4 m	c.314C > G	p.S105X	Fever, diarrhea	Hepato-splenomegaly. Portal hypertension. UGIH	DPM, cholestasis	No	Yes	LT at 6 y 4 m	
		M, 3 m	c.547C > T c.314C > G	p.R183X p.S105X	Neonatal jaundice	Hepato-splenomegaly	Portal widening and fibrosis, ductular reaction	No	Yes	Normalised LFTs on UDCA at 6 y	
		*Sisters* F, 9 y	c.514C > T	p.R172X	Hepato-splenomegaly	Hepato-splenomegaly	DPM	No	Yes	Improved LFTs on UDCA at 11 y	
		F, 4 y			UGIH		DPM, fibro-obliterative loss of bile ducts with DPM	No	Yes	LT at 4 y 8 m	
		M, 3 m	c.379C > T c.314C > G	p.Q127X p.S105X	Neonatal jaundice Fever, cough	Hepato-splenomegaly. Portal hypertension. UGIH	DPM, fibro-obliterative loss of bile ducts with DPM, cholestasis	No	Yes	LT at 1 y 10 m	
Present case	1	F, 59 days	c.667C > T	p.R223X [p.Arg223Ter]	Cholestatic jaundice. Hepato-splenomegaly	Hepato-splenomegaly Anicteric cholestasis	Micronodular cirrhosis, bile ducts proliferation and portal tract abnormalities consonant with DPM or CHF	No	Yes	Mild ↑ LFTs, persistent anicteric cholestasis with preserved protein synthesis, on UDCA and Rifampicin (5y)	Immunofluorescence analysis of primary cilia on cultured skin fibroblasts showed fragmented cilia and centrioles abnormalities

DPM, ductal plate malformation; GGT, serum Gamma glutamyl tranpeptidase; GOT, serum glutamic oxaloacetic transaminase; GPT, serum glutamic pyruvic transaminase; LFT, liver function test; LT, liver transplantation; UDCA, ursodeoxycholic acid; UGIH, recurrent upper gastrointestinal haemorrhage; y, year; m, month; **↑**, increased

Although Fibrocystic Liver Disease, a heterogeneous group of biliary disorders characterized by abnormal development of the embryonic ductal plate, secondary to genetically determined dysfunctions of cholangiocytes cilioproteins, is a well-established cilia-associated liver disorder, isolated and/or severe neonatal cholestasis is rarely recognized as a ciliopathy. However, the recent identification of biallelic mutations in Tetratricopeptide Repeat Domain 26 (TTC26) a well-known ciliary protein, in a rapidly progressive severe neonatal cholestatic condition highlighted the need of including ciliopathies in the differential diagnosis of severe neonatal cholestasis even in the absence of more typical features [16]. Generally, ciliopathies are multisystemic disorders and the liver is frequently involved CHF and CD are the most common liver manifestations of ciliopathies in children. Both the patient’s cohort described by Luan et al. and our case present only hepatic involvement.

Analysis of our patient plasma sterols in addition to increased levels of cholestanol, which is known to be also secondary to chronic cholestasis, showed increased campesterol and sitosterol levels. This was in agreement with the diagnosis of sitosterolemia (OMIM #210250) coincidental with the associated cholestatic pattern due to *ZFYVE19* defect. Although high concentrations of serum phytosterols are correlated with liver disease and severity of cholestasis in Total Parenteral Nutrition-dependent children, liver involvement in sitosterolemia has been (rarely) reported only in adults and never of cholestatic origin [17]. Notably, also the series of Luan patients harbored unexpected variants in several other genes without established relationships with liver disease [5].

## Conclusions

The case here reported offers at least three foods for thought. Firstly, it confirms that ZFYVE19 is a novel cause of high GGT neonatal-onset chronic cholestasis where cilia involvement plays a likely role. The latter specific aspect calls for a continuous monitoring of the patient to verify the liver disease progression (namely, overt sclerosing cholangitis and/or portal hypertension) and whether the phenotype will involve also other organs and systems not yet affected. The coexistence with sitosterolemia, a condition hitherto described having not effects on the liver, also will deserve attention.

Secondly, it further confirms that use of WES may improve the diagnostic yield in children with undiagnosed/undefined cholestasis or compound phenotypes with a likely inheritable background. The application of WES, although not routinely, enables the identification of new causative genes, widening the knowledge on the pathophysiology, eventually resulting in therapeutic implications.

## Supplementary Information

**Additional file 1.** **Supplementary Table 1.** Laboratory monitoring of the patient.

## Data Availability

Data are available by request.

## Abbreviations

CHF: Congenital hepatic fibrosis
DPM: Ductal plate malformation
GGT: Gamma-glutamyltransferase.
GOT: Serum Glutamic Oxaloacetic Transaminase
GPT: Serum Glutamic Pyruvic Transaminase
PFIC: Progressive familial intrahepatic chronic cholestasis
PCHE: Pseudocholinesterase
UDCA: Ursodeoxycholic acid
WES: Whole exome sequencing
