# Supplementary material for: A ZFYVE19 gene mutation associated with neonatal cholestasis and cilia dysfunction: case report with a novel pathogenic variant
Source: Orphanet J Rare Dis. 2021 Apr 14;16:179. doi: 10.1186/s13023-021-01775-8 (PMC8048179; doi:10.1186/s13023-021-01775-8)
Supplement: Supplementary file 1 — Additional file 1. Supplementary Table 1. Laboratory monitoring of the patient. [file 13023_2021_1775_MOESM1_ESM.docx]

| **Supplementary Table 1.** Laboratory data monitoring of the patient | | | | | | | | | | | | | | | | | | | | |
| --- | --- | --- | --- | --- | --- | --- | --- | --- | --- | --- | --- | --- | --- | --- | --- | --- | --- | --- | --- | --- |
|  | **January 2016** | **February 2016** | **March 2016** | **April 2016** | **June 2016** | **July 2016** | **Nov 2016** | **April 2017** | **July 2017** | **December 2017** | **April 2018** | **October 2018** | **February 2019** | **July 2019** | **September 2019** | **March 2020** | **June 2020** | **September 2020** | **November 2020** |  |
| **T.B./**  **D.B.** (mg/dl) | 11.5/6.8 | 6.62/5.78 | 5.18/2.82 | 0.91/0.49 | 0.94/0.29 | 0.74/0.19 | 0.52/0.2 | 0.61/0.23 | 1.32/0.47 | 0.91/0.47 | 0.94/0.42 | 1.03/0.53 | 1,28/0,46 | 1.18/0.54 | 1.06/0.45 | 1.29/0.58 | 1.92/  0.91 | 1.75/0.79 | 1,37/0,56 |  |
| **GPT/**  **GOT** (U/I) | 248/  365 | - | 130/  225 | 85/  97 | 91/  106 | 78/  101 | 74/  109 | 63/  112 | 84/  119 | 126/  139 | 92/  117 | 94/  119 | 89/  135 | 99/  120 | 115/  127 | 146/  175 | 181/  249 | 141/  172 | 130/  155 |  |
| **GGT** (U/I) | - | 1791 | 1137 | 1217 | 769 | 462 | 219 | 279 | 270 | 261 | 286 | 425 | 192 | 249 | 266 | - | 357 | 336 | 392 |  |
| **Albumin** (g/dl) | - | 3.0 | 3.2 | 3.5 | 3.4 | 3.3 | 3.6 | 3.2 | 3.6 | 3.6 | 3.06 | 3.5 | 3.2 | 3.3 | 3.5 | 3.6 | 3.6 | 3.8 | 3,1 |  |
| **PT%** | 100 | 100 |  | 87 | 88 | 78 | 88 | 95 | 88 | 97 | 88 | 100 | 95 | 94 | 101 | 98 | 105 | 92 | - |  |
| **B.A.** (µmol/L) | - | 150 |  |  | 153 |  | 162 |  | 388 | - | - | 502 | 296 | - | - | 300 | 603 | - | 349 |  |
| **Tot Chol** (mg/dl) | 160 | - | 154 | - | - | 149 | 182 | 164 | 216 | 254 | 225 | 230 | 215 | 249 | 275 | 309 | 239 | 235 | 269 |  |
| **Cholestanol** (mg/dl) (nv. < 0.71) | - | - | - | - | - | - | - | - | - | - | - | - | - | 1.44 | - | 1.13 | - | - | - |  |
| **Campesterol** (mg/dl) (nv.< 0.33) | - | - | - | - | - | - | - | - | - | - | - | - | - | 0.89 | - | 1.09 | - | - | - |  |
| **Sitosterol** (mg/dl) (nv < 0.39) | - | - | - | - | - | - | - | - | - | - | - | - | - | 1.40 | - | 1.53 | - | - | - |  |
| **UDCA** (mg/kg/day) | 28 | 28 | 28 | 25.8 | 17 | 20.4 | 20.4 | 21 | 21 | 24 | 24 | 23 | 21,4 | 21,4 | 21,4 | 22 | 23.43 | 23.4 | 25 |  |
| **Rifampin** (mg/Kg/day) | 4.6 | 4.6 | 4.4 | 4.1 | 2.6 | 2.6 | 2.2 | 2 | 0.93 | 0.93 | 0.8 | 0.77 | 0.71 | 0.71 | 0.62 | Stop | 0.62 | 0.62 | 5 |  |
| **Abbreviations. B.A:** bile acids **D.B:** direct bilirubin **GGT:** Gamma-glutamyltransferase. **GOT:**Serum Glutamic Oxaloacetic Transaminase. **GPT:** Serum Glutamic Pyruvic Transaminase. **PT:**prothrombin time **T.B:** total bilirubin. **Tot Cholest.:**Total Cholesterol **T.P:** total proteins, **UDCA:** Ursodeoxycholic acid | | | | | | | | | | | | | | | | | | | | |
